# An Important Announcement

**Published:** 1891-05

**Authors:** 


### An Important Announcement.

FORTHCOMING.

P. Blakiston, Son & Co., the medical publishers of Philadelphia, announce for early publication, "A Handbook of Local Therapeutics," being a practical description of all those agents used in the local treatment of disease, such as ointments, plasters, powders, lotions, inhalations, suppositories, bougies, tampons, etc., and the proper methods of preparing and applying them.

The diseases which chiefly require local treatment are those of the respiratory passages, ear, eye, skin, together with certain general surgical affections, including the diseases of women. In order, therefore, that the various uses of each remedy may be thoroughly set forth, the following gentlemen have assumed the authorship:—Harrison Allen, M.D., emeritus professor of physiology in the University of Penn; laryngologist to the Rush Hospital for consumption; late surgeon to the Philadelphia and St. Joseph's Hospitals. George C. Harlan, M.D., late professor of the diseases of the eye in the Philadelphia Polyclinic and college for graduates in medicine; surgeon to the Willis Eye Hospital, and eye and ear department of the Pennsylvania Hospital. Charles B. Penrose, M.D., surgeon to the German Hospital; instructor in clinical surgery, University of Pennsylvania, and Arthur Van Harlingen, M.D., professor of diseases of the skin in the Philadelphia Polyclinic and College for Graduates in Medicine; late clinical lecturer on dermatology in Jefferson Medical College; dermatologist to the Howard Hospital.

Each remedy will be taken up in alphabetical order, and after a succinct description of their pharmaceutical properties, by Dr. George I. McKelway, will be considered with reference to the local treatment of the affections above outlined. The authors believe that the information contained in this work will not be found elsewhere. The activity in the various lines of special medicine is one of the most striking phases of the times, and has here materially changed many of the older methods of treating disease by local means. The greater part of the literature which appears is not accessible to most physicians. The Handbook, it is believed, will be of value to general practitioners as well as to those who, like themselves, are especially interested in subdivisions of the clinical field.

The work will form a compact volume of about 400 pages, arranged in a manner to facilitate reference and containing, besides the usual index, a complete index of diseases, that will greatly enhance its usefulness.

